# Effect of Polyphenylsulfone and Polysulfone Incompatibility on the Structure and Performance of Blend Membranes for Ultrafiltration

**DOI:** 10.3390/ma14195740

**Published:** 2021-10-01

**Authors:** Tatiana Plisko, Yana Karslyan, Alexandr Bildyukevich

**Affiliations:** 1 Institute of Physical Organic Chemistry, National Academy of Sciences of Belarus, 13 Surganov Street, 220072 Minsk, Belarus; uf@ifoch.bas-net.by; 2 Department of Analytical Chemistry, Petersburg State University, 7/9 Universitetskaya nab., 199034 St. Petersburg, Russia; 3 Argonne National Laboratory, Energy and Global Security Directorate, Chemical & Fuel Cycle Technologies Division, 9700 S Cass Ave, Lemont, IL 60439, USA; Ykarslyan@anl.gov

**Keywords:** membrane, ultrafiltration, polyphenylsulfone, polysulfone, polymer blend, incompability

## Abstract

This study deals with the modification of polyphenylsulfone ultrafiltration membranes by introduction of an incompatible polymer polysulfone to the polyphenylsulfone casting solution to improve the permeability. The correlation between properties of the blend polyphenylsulfone/polysulfone solutions and porous anisotropic membranes for ultrafiltration prepared from these solutions was revealed. The blend polyphenylsulfone/polysulfone solutions were investigated using a turbidity spectrum method, optical microscopy and measurements of dynamic viscosity and turbidity. The structure of the prepared blend flat sheet membranes was studied using scanning electron microscopy. Membrane separation performance was investigated in the process of ultrafiltration of human serum albumin buffered solutions. It was found that with the introduction of polysulfone to the polyphenylsulfone casting solution in N-methyl-2-pyrrolidone the size of supramolecular particles significantly increases with the maximum at (40–60):(60:40) polyphenylsulfone:polysulfone blend ratio from 76 nm to 196–354 nm. It was shown that polyphenylsulfone/polysulfone blend solutions, unlike the solutions of pristine polymers, are two-phase systems (emulsions) with the maximum droplet size and highest degree of polydispersity at polyphenylsulfone/polysulfone blend ratios (30–60):(70–40). Pure water flux of the blend membranes passes through a maximum in the region of the most heterogeneous structure of the casting solution, which is associated with the imposition of a polymer-polymer phase separation on the non-solvent induced phase separation upon membrane preparation. The application of polyphenylsulfone/polysulfone blends as membrane-forming polymers and polyethylene glycol (M_n_ = 400 g·mol^−1^) as a pore-forming agent to the casting solutions yields the formation of ultrafiltration membranes with high membrane pure water flux (270 L·m^−2^·h^−1^ at 0.1MPa) and human serum albumin rejection of 85%.

## 1. Introduction

The application of membrane separation technology has progressed rapidly over the years with the introduction of advanced membranes designed from new materials [1,2]. Membrane separation is beneficial compared to other separation processes due to high selectivity and separation efficiency, no need for the use of additives or chemicals, reducing operating costs as well as minimizing the human health risks [3]. Conventional separation techniques, such as precipitation, crystallization, and centrifugation are known to suffer from poor selectivity of separation, whereas high-resolution techniques, such as chromatography and electrophoresis, are characterized by low yield of product at higher cost [4]. Ultrafiltration is a pressure-driven membrane process which requires the use of porous membranes. Ultrafiltration can be used for water treatment, purification, fractionation, and concentration of the solutions of polymers in microbiology, biotechnology, dairy, food industries, cosmetics, and pharmaceuticals [3,4,5]. Ultrafiltration is known to demonstrate very high process performance and can be fine-tuned to provide high selectivity [5].

Polysulfones, a class of thermoplastic polymers, has become a dominant material in the production of the membranes for separation technology due to low cost and ease of processing, thermal and chemical stability [6]. Polysulfone (PSF) is a widely used material for membrane preparation [7,8,9,10] whereas polyphenylsulfone (PPSU) is a relatively new polymer which features superior properties compared with the more frequently applied PSF and polyethersulfone (PES) [11]. PPSU has better impact strength than PSF and PES [11]. It also has higher chemical resistance—particularly to cleaning and disinfecting agents—and a very low rate of water absorption, making it suitable for applications involving superheated steam sterilization and alkali washing [12,13,14]. PPSU is remarkable for good dimensional stability and excellent resistance to high-energy radiation and heat. All these properties make PPSU a promising candidate for design of novel membranes [11,12,13,14].

To date, the application of PPSU was studied for the development for membranes for ultrafiltration [15,16,17,18,19,20,21,22,23,24,25,26,27,28,29,30,31,32,33], nanofiltration [33,34,35], organic solvent nanofiltration [36,37,38], pervaporation [39,40,41,42,43], gas separation [44,45,46,47], membrane substrate for thin film composite membranes for forward osmosis [48,49], and proton-exchange membrane for fuel cells [50]. PPSU membranes were revealed to be effective in water treatment [33]: heavy metal [17,26], toxic dyes [18,20], humic acids [25] removal from aqueous solutions [17,26], and decontamination of arsenic from drinking water [21,22,23]. PPSU membranes were developed and investigated for pervaporation separation of biobutanol form ABE mixture [39], dehydration of acetic acid [40,41,42] and alcohols [43] via pervaporation, biogas upgrading [44] and hydrogen purification [45,46].

However, no examples of the application of membranes based on PPSU in industry have been found, which is associated with the low flux of the obtained materials [26,31]. For instance, pristine ultrafiltration PPSU membranes prepared via non-solvent induced phase separation (NIPS) demonstrate very low pure water flux ranging from 0 to 10 L∙m^−2^∙h^−1^ at 0.1 MPa [26]. The reason for the low flux of PPSU membranes is the restriction of the variation of casting solution composition due to low miscibility of PPSU with commonly used pore-forming additives (polyethylene glycol (PEG), polyvinypyrrolidone (PVP)) in aprotic amide solvents (N-methyl-2-pyrrolidone (NMP), N,N-dimethylformamide (DMF) etc.) [27,28]. This study is focused on the modification of PPSU ultrafiltration membranes to increase their permeability.

The PPSU membranes have to be modified to improve their separation performance, hydrophilicity and antifouling resistance for practical applications. One of the methods to improve membrane separation and antifouling performance is blending—the introduction of additives into the casting solution. Blending is a simple, reproducible, and an effective way of membrane structure modification. The effect of the addition of hydrophilic water-soluble polymers and oligomers to the PPSU casting solution [16,17,18,27,28,31,51], surfactants [31], low molecular weight substances [31], inorganic nanoparticles [17,18,19,21,22,23,34,51,52,53,54], and metal organic frameworks [37] were investigated and reported.

However, the disadvantage of adding water-soluble polymers, oligomers and surfactants to the casting solution is that they are easily washed out during membrane preparation process and membrane operation [55]. The disadvantage of using nanoparticles as an additive to the casting solution for membrane preparation is low compatibility and low adhesion with polymer membrane matrix as well as nanoparticle agglomeration which leads to the formation of microdefects in membrane selective layer. Microdefects can deteriorate membrane performance. Moreover, membrane modification by blending nanoparticles is technologically complex due to the additional step of uniform dispersion of nanoparticles in the casting solution. Nanoparticle blending features low reproducibility because membrane properties are determined by the uniformity of the dispersion of nanoparticles in membrane matrix.

Another promising approach of PPSU membrane modification is an addition of second membrane-forming polymer to PPSU casting solution which yields preparation of blend membranes. PPSU/PES [19,20], PPSU/cellulose acetate derivatives [21,22], PPSU/cellulose acetate [23], PPSU/polyetherimide [25], PPSU/PSF [26,56], sulphonated PPSU/polybenzimidazol [45], PPSU/poly(bisphenol A-co-4-nitrophthalic anhydride-co-1,3- phenylenediamine) (PBNPI) [46], PPSU/polymer of intrinstic microscopy (PIM) [47] blend membranes were developed and investigated for ultrafiltration and gas separation. The addition of a second polymer to the casting solution could affect both the physical and the chemical properties of the final membrane. For instance, the solution viscosity, phase separation behavior, pore size and pore size distribution, morphology, hydrophobicity, separation performance, and mechanical strength can be adjusted. It was found that the addition of the second polymer results in the combination of “polymer-polymer” phase separation and “liquid-liquid” phase separation upon membrane preparation by non-solvent induced phase separation (NIPS). It allows changing structure of membrane selective layer. Polymer blending method gives an opportunity to obtain a high flux, high selectivity, and low fouling membrane with desirable mechanical and thermal properties [19].

The effects of the blending ratio, activated carbon, and polyethylene glycol (M_w_ = 200 g·mol^−1^) addition on membrane structure and performance were studied in the PPSU/polyetherimide system [25]. According to the results, hydrophilicity and pore size increased after blending PPSU with polyetherimide, PEG, and activated carbon. The increase in hydrophilicity had a favorable effect on the permeate flux and membrane fouling resistance to humic acids [25].

PPSU/PSF blend membranes with beta glycine and PEG addition to the casting solution were studied [26]. The authors used these blend membranes for bovine serum albumin (BSA) solution ultrafiltration and heavy metal removal from water. The addition of PEG-1000 (M_n_ = 1000 g·mol^−1^) caused a significant increase in hydrophilicity, whereas bio-resistance increased from 50% to 73%. These membranes were also shown to have rejections at 99.48% and 95.5% for Pb^2+^ and Cd^2+^, respectively, and high pure water flux of 65.88 L·m^−2^·h^−1^ at 0.5MPa [26].

The objective of this work was to study the correlation between the properties (phase state, structure, and viscosity) of the blend PPSU and PSF solutions and properties (structure, performance in ultrafiltration) of porous anisotropic membranes obtained via NIPS. The advantage of this study is the thorough and detailed investigation of the effect of addition of the second incompatible polymer on the structure and peculiarities of phase separation of PPSU solutions in NMP. The blend PPSU/PSF solutions were investigated using turbidity spectrum method, optical microscopy, the measurements of dynamic viscosity and turbidity.

It is widely known that supramolecular particles (SMP) are temporally existing agglomerates of macromolecules in polymer solutions which are continuously destroyed by thermal motion. To characterize the supramolecular structure of the polymer solutions, the turbidity-spectrum method was applied [57,58,59]. This method makes it possible to find the average diameter and concentration of supramolecular particles (SMPs) in a polymer solution without subjecting the system to any external action. The turbidity spectrum method is based on the rigorous theory of scattering on spherical particles (Mie theory) [57,58,59]. In turbidity spectrum method polymer solution with the complex structure is modeled by colloid system in which the dispersed phase is assumed to be the ensemble of SMPs and dispersion medium is considered to be the true solution of macromolecules. This method can be used when more light energy is scattered on macromolecule agglomerates (SMP) compared to the molecular scattering (on concentration fluctuations) [57].

The structures of the prepared PPSU/PSF blend flat sheet membranes were studied using scanning electron microscopy. Membrane separation performance was studied in the ultrafiltration of human serum albumin (HSA) solutions. The investigation of the structure, physical-chemical properties, and peculiarities of the phase separation of the blend PPSU/PSF solutions in NMP allowed reaching considerably higher water flux than previously achieved for these systems maintaining high HSA rejection coefficients due to the increase in porosity and decrease in the thickness of the selective layer of cast membranes. The application of PPSU/PSF blends as membrane forming polymers and PEG-400 as a pore-forming agent allowed transforming impenetrable membranes to high-flux membranes.

## 2. Materials and Methods

### 2.1. Materials

**Figure 1:**
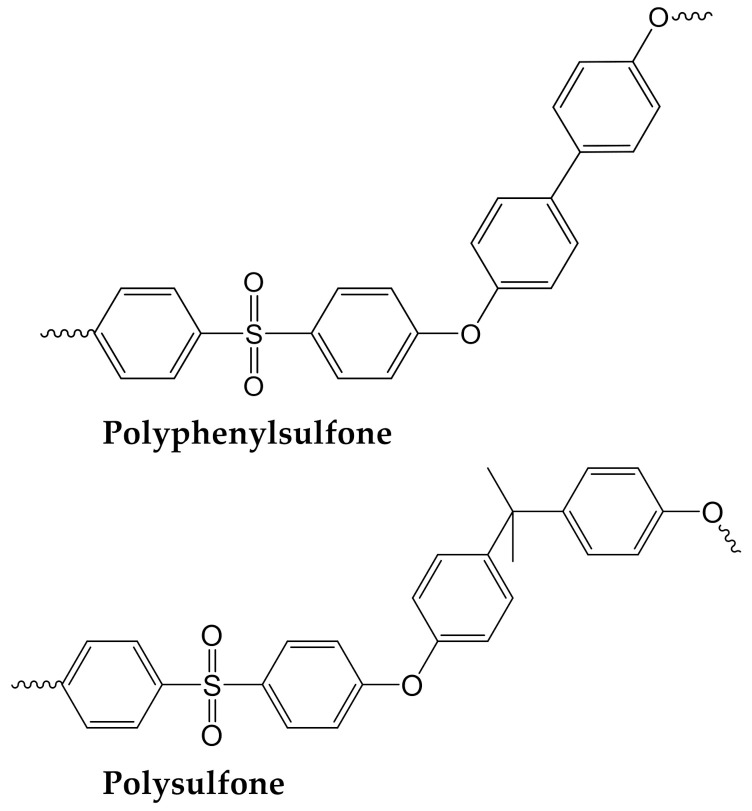
Chemical structure of the polymers used in this study.

Polyphenylsulfone (PPSU, Ultrason P 3010, BASF, Ludwigshafen, Germany, M_w_ = 4.8 × 10^4^ g∙mol^−1^) and polysulfone (PSF, Ultrason S 6010, BASF, M_w_ = 4.5–5.5 × 10^4^ g∙mol^−1^) were used for polymer solution and membrane preparation. The structures of the polymers are shown in Figure 1. Polysulfones were dried at 120 °C for 8 h in a drying oven prior to processing. PES Ultrason E 6020P PES (Mn = 1.1 × 10^4^ g∙mol^−1^ , M_w_ = 5.8 × 10^4^, BASF, Germany) was applied for the preparation of 1 wt.% solutions in N-methyl-2-pyrrolidone (NMP) to determine the coagulation values of different non-solvents.

Polyethylene glycol (PEG-400, BASF, Ludwigshafen, Germany) with M_n_ = 400 g∙mol^−1^ was used as the additive to the polymer solution, and NMP (EKOS-1, Russia) was utilized as the solvent without any further purification.

Human serum albumin (HSA, M_w_ = 6.7 × 10^4^ g∙mol^−1^, isoelectric point of pH 4.7) was supplied by Sigma Aldrich. Anhydrous sodium monobasic phosphate (KH_2_PO_4_) and sodium dibasic phosphate trihydrate (K_2_HPO_4_∙3H_2_O) were purchased from Merck and used for the preparation of phosphate buffer solutions (0.05 M, pH = 7.0). MilliQ water was used throughout this study.

### 2.2. Preparation of Polymer Solutions

Blend PPSU/PSF solutions and solutions of pristine polymers (PSF and PPSU) in NMP with the total polymer concentration of 20 wt.% were prepared. PPSU to PSF weight ratio was in the range from 10:90 to 90:10 with the step of 10 wt%. PEG-400 was used as an additive to the solution in some cases. The properties of the blend PPSU/PSF solutions were studied and they were used for the casting of flat sheet membranes. The compositions of the solutions and membranes are listed in the Table 1. Polymer solutions were prepared by dissolving 20 wt% of polymer pellets in NMP under continuous stirring using overhead stirrer (IKA RW 20 Digital, Germany) at the rotation speed of 500–700 rpm for 4 h at 120 °C in a sealed container to ensure no moisture was absorbed. The PEG-400 was dissolved together with PPSU. The casting solutions were left for 24 h to remove any entrapped air bubbles. For solution preparation, all glassware was rinsed with distilled water and dried prior to usage to remove any dust collected.

### 2.3. Investigation of Polymer Solutions

#### 2.3.1. Viscosity Measurements

A Brookfield DV III-Ultra rotary viscometer was used to measure the dynamic viscosity (η) of polymer solutions at the shear stress of 20 N·m^−2^. Measurements for each of the solution were carried out at 25.0 °C. Three different samples of the polymer solutions were studied and the average viscosity was calculated. It was found that the relative error does not exceed 2%.

#### 2.3.2. Turbidity Measurements

The measurements of the turbidity of the polymer solutions were performed using a 2100 AN turbidimeter produced by HACH (Düsseldorf, Germany) with a tungsten filament lamp as a light source and a filter with a wavelength of 860 nm. The turbidimeter is calibrated in Nephelometric Turbidity Units (NTU) using formazine aqueous solutions.

#### 2.3.3. Determination of the Average Diameter of Supramolecular Particles (SMP) in the Polymer Solutions

The absorbance spectrum D(λ) of the polymer solutions was determined using a Metertech UV–VIS SP 8001 spectrophotometer in the wavelength range 400–800 nm. Then the dependence of the logarithm of absorbance (lg*D*) on the logarithm of wavelength (lg*λ*) was plotted. Due to the fact that turbidity spectra in logarithmic coordinates have a rather small curvature (according to the Angström formula, *τ* = *Aλ^−n^* for a limited wavelength interval [57]), the tangent is drawn using the least-squares method (LSM), by the technique used for drawing a straight line through the given points [57]. The tangent of the plot (∆lg*D*/∆lg*λ*) is propotional to the diameter of the particles which scatter the light (Equation (1)).

(1) $$n=-\frac{\Delta \mathrm{lg}D}{\Delta \mathrm{lg}\lambda }$$

The refractive indices of the polymer solutions were measured on an RL 3 laboratory refractometer (Poland), thermostated at T = 20.0 °C. According to the refractometer measurements the relative refractive index m was calculated using the Equation (2):

(2) $$m=\frac{\mu }{\mu_{0}}$$

where *μ*—refractive index of the SMP of the dispersed phase (polymer or polymer blend); *μ*_0_—refractive index of the dispersion medium (polymer solution).

The refractive index of the polymer blend, *n_tot_*, was calculated according to the additive scheme (Gladstone-Dale law) from the refractive indices of the polymers constituting the blend with correction made for their ratio according to the Equation (3) [60]:

(3) $$n_{tot}=n_{1}\varphi_{1}+n_{2}\varphi_{2}+\ldots +n_{N}\varphi_{N}$$

where *n*_1_, *n*_2_, and *n_N_* are the refractive indices of the blend components and *φ*_1_, *φ*_2_, and *φ_N_* are the volume fractions of the components in the blend.

According to Dizman et al. [6] the refractive index of PSF is 1.63 and the refractive index of PPSU is 1.67. In our case, using the refractive index of the polymer is a fairly rough approximation as SMP are the most structurally perfect fragments of polymer crystallites which can contain solvent molecules and the compositions of the SMPs in the blend solutions are not known exactly.

According to the calculated values of m and n using the characteristic function of scattering of monodisperse systems tabulation, the values of K(α, m) and α were determined [57]. K(α, m) is the coefficient of scattering of one particle or efficiency scattering factor, α—is geometrical (shape) factor of the particle.

The average diameter of SMP (*r_w_*) was calculated according to the Equation (4):

(4) $$r_{w}=\frac{\alpha \cdot \lambda_{m}^{\prime }}{2\pi }$$

where

(5) $$\lambda_{m}^{\prime }=\frac{\lambda_{m}}{\mu_{0}}$$

where *λ_m_*—the mean wavelength in the studied range of 400–600 nm, *λ_m_* = 500 nm.

The concentration of the SMP in the solution (*N*, the number of SMP in 1 cm^3^) was calculated according to the Equation (6):

(6) $$N=\frac{1.26\cdot 10^{17}\cdot \tau }{{\left(\lambda_{m}^{\prime }\right)}^{2}\cdot K\left(\alpha ,m\right)\alpha^{2}}$$

where *τ* is turbidity.

(7) $$\tau =\frac{2.3D}{l}$$

#### 2.3.4. Optical Microscopy Studies

To study the structure of solutions based on pristine PPSU and PSF, as well as blend solutions of PPSU/PSF mixtures in NMP, an OLYMPUSGX41 metallographic inverted microscope (Japan) was used. It is designed to operate in a bright field and with polarization. To prepare the samples, a thin film of the polymer solution was applied to a glass slide and covered with a cover glass. To study the evolution of the structure of polymer solutions due to the water sorption from air and slow precipitation the optical microscopy images were taken at the beginning of the experiment and in 6, 12, 18 and 24 h.

#### 2.3.5. Determination of Coagulation Values of Polymer Solutions

Coagulation values (CV, g∙dL^−1^) of different non-solvents (water, PEG-400, glycerol, ethylene glycol, isopropanol) for PPSU and PSF solutions in NMP were determined as the quantity (g) of the non-solvent needed to cause the phase separation of 100 mL (1 dL) of 1 wt.% PPSU or PSF solution by titration method (cloud point method) described in [27].

### 2.4. Preparation of Membranes

Flat sheet asymmetric PPSU/PSF blend membranes were prepared via non-solvent induced phase separation (NIPS). Compositions of the casting solutions for membrane preparation are summarized in Table 1. Flat sheet membranes were prepared by casting a polymer solution on a clean and dry glass plate using a special casting knife with a thickness of 150 μm. The obtained polymer film was immersed immediately in a non-solvent bath (MilliQ water at T = 20 °C). Polymer precipitation occurs due to the contact of polymer solution with the non-solvent (water), and the membranes are formed. All procedures were performed in an air-conditioned room with a constant temperature and constant value of relative humidity (T = 20 °C; H_r_ = 70%). After 30 min, the membranes were placed in a fresh distilled water bath and left there for 24 h to ensure sufficient removal of solvent and stability of the final structure of the membranes. Finally, the membranes were stored in MilliQ water for further analysis.

### 2.5. Membrane Characterization

#### 2.5.1. Scanning Electron Microscopy Studies

Prior to scanning electron microscopy (SEM) studies, the membranes were impregnated with 50 wt% glycerol aqueous solution and then dried at room temperature for 72 h. The structure of the flat sheet membranes was studied using a LEO 1420 scanning electron microscope. Cleaved cross-sections of the membrane samples were prepared by cryogenic fracture in liquid nitrogen followed by gold coating using cathode sputtering in an EMITECH K 550X vacuum system.

#### 2.5.2. Pure Water Flux Measurements

Ultrafiltration experiments were performed using a stirred ultrafiltration cell with 200 ml processing volume and effective membrane area of 24.6 cm^2^. Membrane samples were placed in the test cell with the selective layer facing the feed. To condition the studied membranes and achieve stationary flux MilliQ water was filtered through the membranes at 0.2 MPa for 1 h. These pre-conditioned membranes were used in subsequent filtration experiments at 0.1 MPa. Pure water flux (PWF) was determined at a transmembrane pressure of 0.1 MPa after 30 min of ultrafiltration. PWF was determined as follows:

(8) $$J=\left(\frac{V}{S\cdot t}\right)$$

where, *J* is the flux (L m^−2^ h^−1^), *V* is the permeate volume (L), *S* is the membrane surface area (m^2^), and *t* is the permeation time (h).

#### 2.5.3. Study of Membrane Performance in Human Serum Albumin Solution Ultrafiltration

After pure water flux determination, the ultrafiltration cell was filled with 0.1 wt.% human serum albumin (HSA) solution in the phosphate buffer (0.05 M, pH = 7.0). Permeate was collected over 30 min intervals and protein flux was calculated according to the Equation (8). The protein contents were analyzed with UV-Vis spectrophotometer (Metertech UV-Vis SP 8001) at a wavelength of 280 nm. The rejection (R, %) was calculated using the Equation (9):

(9) $$R=\left(1-\frac{C_{p}}{C_{f}}\right)\cdot 100\%,$$

where *C_p_* and *C_f_* are the HSA concentrations in permeate and feed.

## 3. Results and Discussions

### 3.1. Properties of PPSU/PSF Blend Solutions

#### 3.1.1. Studies of Viscosity and Coagulation Values

Solutions of PPSU, PSF, and their blends in NMP were found to demonstrate low turbidity level (1.4–1.6 NTU). It was found that the turbidity of blend solutions does not change when PPSU to PSF ratio increases. It was shown that these solutions are stable for a long time regardless of the PPSU/PSF ratio.

**Figure 2:**
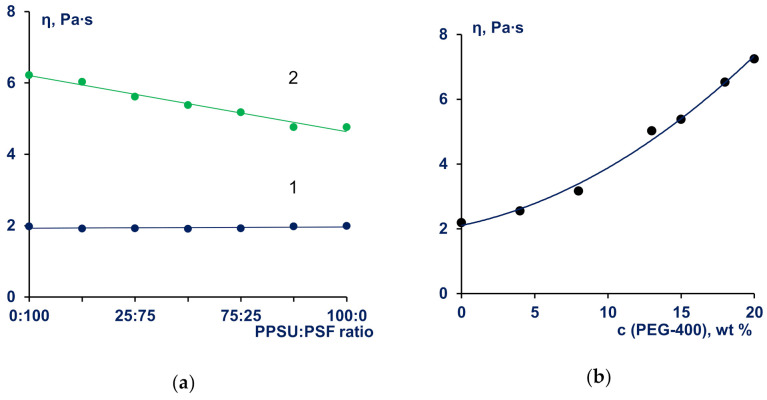
Viscosity of PPSU/PSF blend solutions in NMP: (**a**)—dependence of dynamic viscosity (η) of 20 wt% polymer solutions in NMP on PPSU/PSF blend ratio, PEG-400 concentration: 1–0 wt%; 2–15 wt%; (**b**)—variation of the viscosity of the PPSU/PSF/PEG-400/NMP solutions with PEG-400 concentration. PPSU/PSF blend ratio is 50:50.

Dynamic viscosity of the PPSU/PSF blend solutions with total polymer concentration of 20 wt.% was shown to be very close to the viscosity of the 20 wt.% solutions of pristine polymers over the whole range of PPSU to PSF ratio (1 in Figure 2a). When PEG-400 is added to the solution the system changes from “polymer-solvent” to “polymer-solvent-non-solvent”. This transfer yields the significant increase in the viscosity of 20 wt% solutions of PPSU, PSF, and their mixtures in NMP (Figure 2b).

Cloud points of the 1 wt.% PPSU, PSF, and PES solutions in NMP upon titration by various coagulants were determined and coagulation values (CV) were calculated (Table 2). PEG-400 is known to be a weak coagulant for polysulfones. From Table 2 it can be concluded that CV determined for a number of coagulants are higher for PPSU in NMP than for PSF. CV distinguishes the precipitation power of the coagulant with respect to the polymer solution. When CV is high, it means that the coagulant is weak. On the other hand, CV can also be applied as a characteristic of the thermodynamic quality of the solvent with respect to the dissolved polymer. For instance, if two different polymers are dissolved in the same solvent, the comparison of the CV of the same coagulant for these two polymer solutions can provide the information on the solvent quality for these polymers. The higher the CV in this case the better the solvent quality for the polymer.

However, PES has the highest CV in NMP compared to PPSU and PSF. The viscosity of 20 wt% PPSU-15 wt% PEG-400 solution (4.8 Pa∙s) in NMP is considerably lower than the viscosity of 20 wt% PSF -15 wt% PEG-400 solution (6.2 Pa∙s), despite both PPSU and PSF having close molecular weights, i.e., PPSU Ultrason P 3010 (M_w_ = 4.8 × 10^4^ g∙mol^−1^), PSF Ultrason S 6010 (M_w_ = 4.5–5.5 × 10^4^ g∙mol^−1^).

It is known that viscosity of concentrated solutions of semi-rigid-chain polymers is higher when solvent quality is lower; hence, the difference in coagulation values for PPSU and PSF solutions in NMP means that solvent quality of NMP is higher for PPSU than for PSF (Table 2). Table 2 shows that NMP demonstrates the highest solvent quality with respect to PES compared to other polysulfones, and the lowest—for PSF. PPSU takes the middle position between PES and PSF regarding the solvent (NMP) quality.

It was found that the viscosity of PPSU/PSF blend solutions in a bicomponent solvent mixture NMP/PEG-400 changes additively depending on the PPSU/PSF ratio and decreases when PPSU amount increases (Figure 2a). Increase in the PEG-400 concentration in 50:50 PPSU/PSF blend solution from 0 wt% up to 20 wt% yields more than three-fold viscosity increase from 2.2 to 7.25 Pa∙s (Figure 2b).

It is known that for semi-rigid polymers, introduction of a non-solvent (PEG-400) to the solution leads to the decrease in solvent quality and causes increase in the viscosities of the concentrated polymer solutions. This dependence is individual for various coagulants and depends on their coagulation values. As follows from Table 2, PEG-400 is a softer coagulant for PPSU than for PSF, and that is why PPSU/NMP/PEG-400 solution viscosity is lower than the viscosity of PSF solution with the same composition and concentration.

#### 3.1.2. Structure Studies of PPSU/PSF Blend Solution

**Figure 3:**
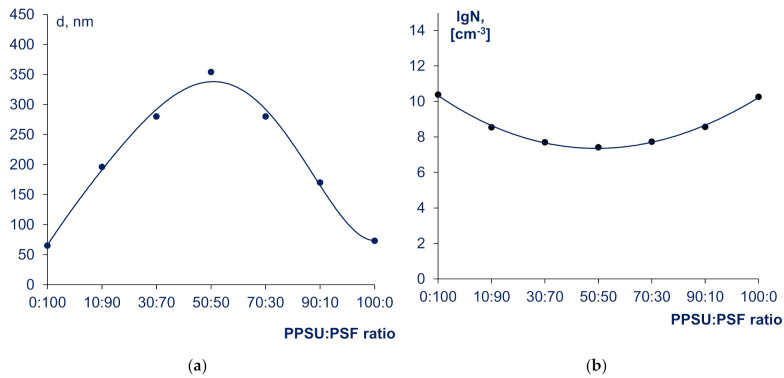
Average diameter (d) (**a**) and logarithm of number (lgN) (**b**) of supramolecular particles (in 1 cm^−3^ of solution) vs. the PPSU:PSF blend ratio in the solution with total concentration of polymers 20 wt%.

The average diameter (d) and concentration of supramolecular particles (SMPs) (N) was calculated using the turbidity spectrum method (Figure 3). The smallest SMPs and the highest concentration of SMPs was revealed to be in 20 wt% solutions of pristine PPSU and PSF (Figure 3a). It was found that the average diameter of PPSU SMPs (76 nm) is bigger than the average diameter of PSF SMPs (65 nm), whereas the concentration of PPSU particles is 1.3 times lower than concentration of PSF particles (1.8 × 10^10^ and 2.4 × 10^10^ cm^−3^, respectively) (Figure 3). This fact corroborates that PPSU tends to form more polymer-polymer interactions in NMP than PSF. This can be due to higher hydrohobicity and higher macromolecular chain rigidity of PPSU compared to PSF [6].

It was revealed that for PPSU:PSF blend solutions in NMP, there was an increase in average diameter of SMPs (up to 196–354 nm) and a decrease in their number (2.6 × 10^7^–3.3 × 10^8^ cm^−3^) by two to three orders of magnitude occur compared to the solutions of pristine polymers in NMP. Ten percent addition of PSF in PPSU was found to increase of SMPs average diameter from 65 to 200 nm. In turn, ten percent addition of PPSU in PSF changes supramolecular particle size from 76 to 150 nm. Overall, dependence of SMP average diameter and concentration on the ratio of PPSU:PSF has extremum points: the biggest SMP diameter and the lowest concentration are observed for polymer ratios close to equivalent (PPSU:PSF = 50:50).

##### Optical Microscopy Studies

**Figure 4:**
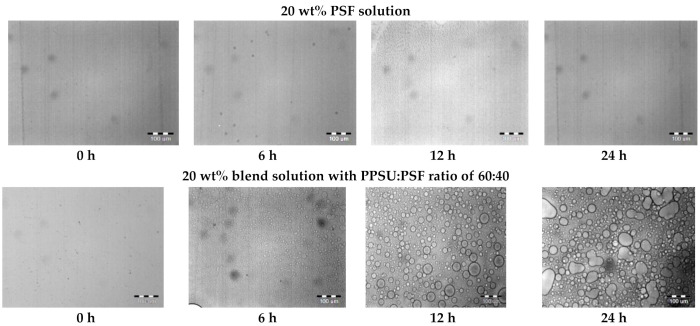
Optical microscopy photographs of PSF and PPSU:PSF = 60:40 blend solutions at the moment of placement and after keeping them for 6, 12, and 24 h between two glass slides.

As mentioned above blend PPSU/PSF solutions in NMP are homogeneous and transparent. Optical microscopy studies reveal the changes in the structure of the blend solution, when a thin film of the solution is placed between two microscope glass slides. Examples of optical microscopy photographs of pristine PSF solution in NMP and 60:40 PPSU-PSF blend solution exposed in the air for 24 h at 60% relative humidity are shown in Figure 4. It can be seen that the degree of heterogeneity increases upon exposing the PPSU/PSF blend solution placed between two microscope glass slides for 6 h in the air. Further exposure results in the formation of emulsion of one polymer in another. At the same time, no such effect is observed for the solutions of pristine polymers. The structure of thin films of pristine PSF and PPSU solution in NMP remains unchanged even after 24 h of exposure in the air.

It was found that when thin films of the solutions are exposed to the air, their structure is revealed as a result of water adsorption and slow precipitation. Accordingly, though PPSU and PSF have similar chemical structure, their solutions in NMP form emulsion of one polymer solution in another polymer solution, and hence these polymers are incompatible. The similar results were obtained in our previous work focused on the study of the effect of the solvent on the properties of solutions of PSF/PES blends in NMP and N,N-dimethylacetamide [61]. It was established using optical microscopy that the PSF and PES blend solutions in both solvents are complex two-phase systems. These are emulsions of two visco-elastic liquids, in which the dispersed phase and the dispersing medium are concentrated solutions of polymers. Furthermore, PSF and PES blend solutions in NMP constitute a size-homogeneous system consisting of interpenetrating domains of PSF and PES solutions, in which it is impossible to distinguish neither the dispersed phase, nor the dispersing medium [61]. However, when using N,N-dimethylacetamide as a solvent, PSF and PES form an emulsion of one polymer solution in the phase of the other one with droplets of the dispersed phase that significantly differ in sizes.

Due to close values of the refraction indices of PPSU and PSF, the studied PPSU/PSF blend solutions are visually transparent, and optical microscopy detects no change in the structure without preliminary exposure to air.

This approach, consisting in exposing the polymer film to the air between two microscope slides before taking photographs, was used to study the structure of 20 wt% solutions of diverse component ratios. The exposure time for all the thin solution films was 18 h.

**Figure 5:**
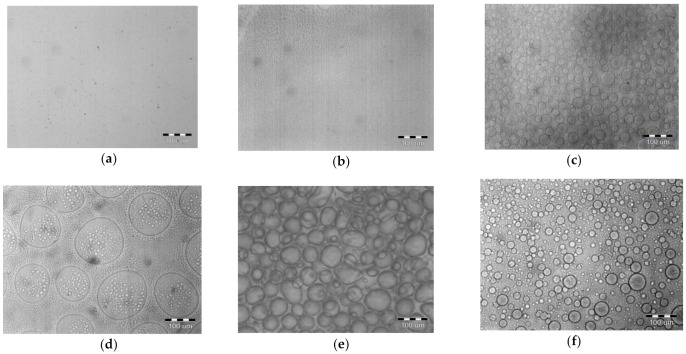

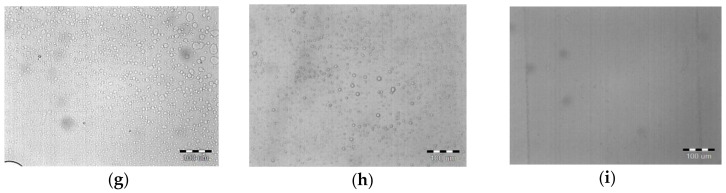
Optical microscopy photographs of PPSU/PSF blend solutions in NMP after keeping 18 h between two glass slides, PPSU:PSF blend ratio: (**a**)—0:100; (**b**)—10:90; (**c**)—30:70; (**d**)—40:60; (**e**)—50:50; (**f**)—60:40; (**g**)—70:30; (**h**)—90:10; (**i**)—100:0.

The data obtained are illustrated on Figure 5. It was revealed that PPSU/PSF solutions are two-phase heterogeneous systems—emulsions of the solutions of one polymer in the solution of another (Figure 5b–h). Their structures are completely different from the structures of the solutions of pristine PPSU and PSF (Figure 5a,i). It was revealed that the sizes of the droplets of the dispersed phase significantly change depending on the polymer ratios (Figure 5b–h). Blend solutions with PPSU to PSF blend ratios (30–70): (70:30) were found to have broad droplet size distribution (Figure 5c–g). The largest droplets are detected in (40–60):(60–40) PPSU:PSF solutions which is in complete agreement with the results obtained by turbidity spectrum method (Figure 3). It was found that the droplet size distribution is the widest for these PPSU:PSF ratios. The blend solution with a PPSU:PSF ratio of 40:60 was revealed to be a multiple emulsion. This multiple emulsion contains spherical droplets consisting of one phase and smaller droplets of another phase included in bigger ones (Figure 5d). When PPSU to PSF ratio is 50:50 the polymer system consists of interpenetrating emulsion droplets and it is difficult to identify dispersed phase and dispersing medium (Figure 5e). It was found that the solutions with the content of the second incompatible polymer of 10 wt% in the blend feature the smallest emulsion droplets and the narrowest droplet size distribution (Figure 5b,h).

It can be concluded that PPSU and PSF are thermodynamically incompatible polymers that is proved by the heterogeneity of their blend solutions. It was found that the degree of heterogeneity is the highest for solutions close to equivalent PPSU:PSF ratios (40–60):(60–40). This fact is confirmed by optical microscopy studies, as well as by the turbidity spectrum method.

### 3.2. Effect of PPSU-PSF Blend Ratio on the Structure and Performance of Ultrafiltration Membranes

Three membrane series were prepared via non-solvent induced phase separation (NIPS) based of PPSU/PSF blend solutions in NMP: A—based on 20 wt% PPSU/PSF blend casting solutions of different ratios; B—from 20wt% PPSU/PSF casting of different ratios with the addition of 15 wt% PEG-400 as a non-solvent; and C—the membranes with the PPSU:PSF blend ratio 50:50 and PEG-400 concentration 5 wt%, 10 wt%, 13 wt.%, 15 wt%, 18 wt%, 20 wt% (Table 1). The membrane structure was studied by SEM. Pure water flux, HSA solution flux and HSA rejection coefficient were also determined for all prepared membranes.

#### 3.2.1. Membrane Structure Investigation by SEM

**Figure 6:**
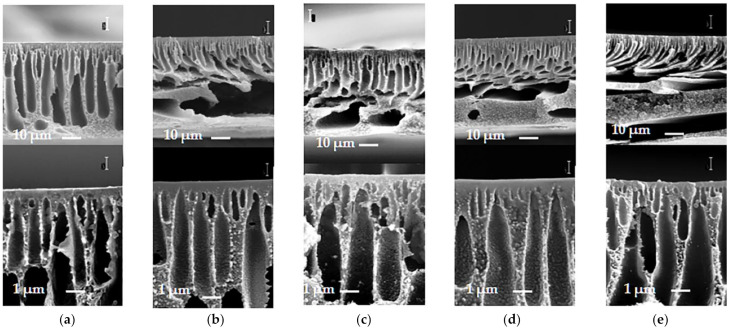
SEM images of the membrane cross-section and enlarged fragment of cross-section, PPSU:PSF ratio in the casting solution: (**a**)—0:100 (PSF100); (**b**)—30:70 (A30); (**c**)—50:50 (A50); (**d**)—70:30 (A70); (**e**)—100:0 (PPSU100).

SEM images of the membrane’s cross-sections and enlarged fragments of the cross-section in the vicinity of the selective layer (series A) are shown on Figure 6. It was shown that the prepared membranes have clearly visible asymmetric structure. Membranes feature a thin selective layer, which determines their transport properties in ultrafiltration (permeability and selectivity). Next to the selective layer, the transitional layer and the drainage layer are located (Figure 6). Drainage layer is known to serve as a substrate and provides mechanical strength to the membrane. Drainage layer is a sponge-like matrix pierced by wedge-shaped macrovoids. The macrovoids of the drainage layer in all membrane samples look like elongated channels which widen from the top membrane side to the periphery of a membrane. There are large macrovoids in the deepest membrane layer in PPSU and blend PPSU-PSF membranes (A30, A50 and A70), which are oriented along the membrane’s surface. The presence of large macrovoids in the membrane drainage layer is known to reduce membrane tensile strength. However, this problem is usually solved via the reinforcement of commercial flat sheet membranes by polyether or polypropylene non-woven support to increase their mechanical strength and ability to withstand pressure. It is worth noting that nowadays all commercial ultrafiltration membranes are produced by casting the polymer solution on non-woven support.

**Figure 7:**
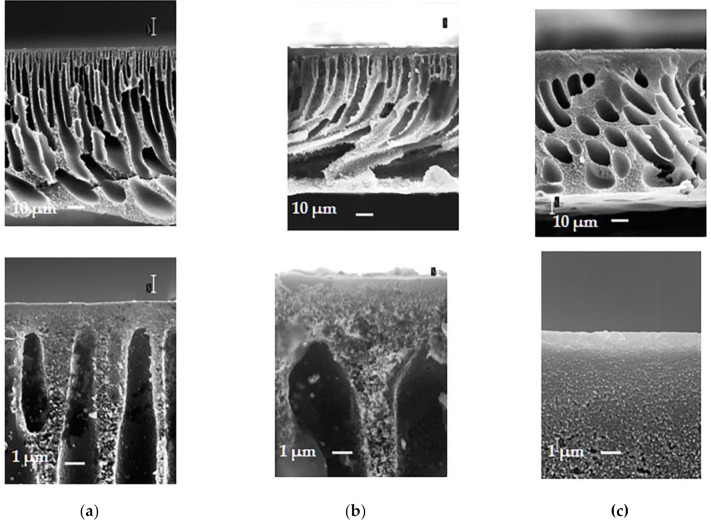
SEM-images of the cross-section and enlarged fragment of cross-section (selective layer) of membranes prepared from a polymer solution with the addition of 15 wt% PEG-400 in NMP (series B), PPSU:PSF ratio: (**a**)—0:100 (PSF-PEG); (**b**)—50:50 (B50); (**c**)—100:0 (PPSU-PEG).

It was found that the addition of PEG-400 to the casting solution yields the reduction of the thickness of the selective layer of the membranes (Figure 7). It is clearly visible that, similar to a series of membranes without addition of PEG-400, the selective layer of the membrane cast from the solution with PPSU:PSF ratio of 50:50 (Figure 7b) feature highly porous spongy structure. The substrate layer of the membrane is permeated with wedge-shaped macrovoids and has a well developed surface, which reduces the resistance to the flow of the feed solution and increases membrane’s flux. The sample of the membrane cast from 20 wt% PPSU solution is structurally different from that cast from PSF and PPSU/PSF blend, and its drainage layer has a sponge-like structure with teardrop-shaped pores. The selective layer of the membranes is dense and features low porosity.

#### 3.2.2. Study of the Separation Performance of Blend Membranes

**Figure 8:**
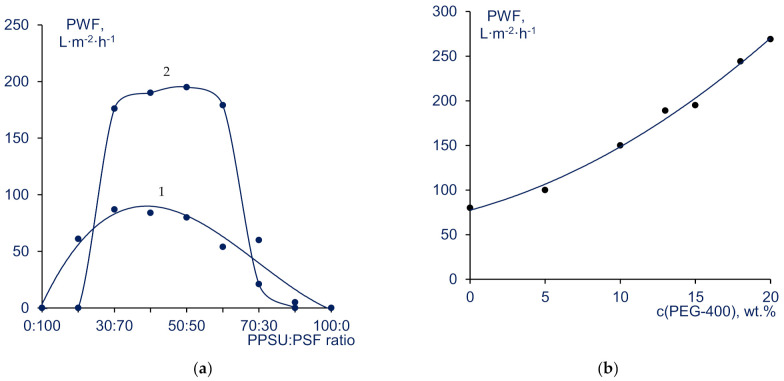
Pure water flux (PWF) of blend PPSU-PSF membranes. (**a**)—dependence of PWF of membranes on the PPSU:PSF blend ratio, PEG-400 concentration in the casting solution: 1–0% PEG-400 (series A); 2–15 wt% PEG-400 (series B); (**b**)—variation of PWF of membranes (ratio of PPSU:PSF is 50:50) with PEG-400 concentration in the casting solution.

It was found that the pure water flux (PWF) of membranes cast from 20 wt% PPSU-PSF blend solutions is significantly different from membranes made of pristine PSF or PPSU solutions. It was found that neither PPSU100, nor PSF100 membranes demonstrate hydraulic permeability. As shown on Figure 8a (curve 1), PWF increases drastically for membranes cast from PPSU/PSF blends compared with the membranes prepared from pristine PPSU and PSF. The addition of 10 wt% PPSU to PSF was shown to increase PWF from 0 to 54 L∙m^−2^ h^−1^ (1 in Figure 8a). As for PPSU, a significant increase in PWF is observed when at least 30 wt.% of PSF is added to the PPSU/PSF blend (at 70:30 ratio). It was revealed that membranes cast from (30–50):(70–50) PPSU:PSF solutions are the most permeable and demonstrate PWF values as high as 80–87 L∙m^−2^ h^−1^. These ratios are in a good agreement with the data obtained by optical microscopy studies of casting solutions and the values obtained by turbidity spectrum method. It is worth noting that PPSU/PSF blend membranes with the highest PWF are obtained from the casting solutions with the biggest emulsion droplets when exposed in air and biggest average diameter of SMPs (Figure 3 and Figure 5). When polymer membrane is prepared via NIPS the membrane structure is formed due to the liquid-liquid phase separation (separation in to the highly concentrated polymer solution and low concentrated polymer solution) due to the contact of polymer solution with the non-solvent (water). The PPSU-PSF incompatibility results in another mechanism of phase separation upon membrane preparation: liquid-liquid phase separation is accompanied by the polymer-polymer (PPSU-PSF) separation. It leads to the formation of the more porous structure of the membrane selective layer resulting in higher pure water flux. Thus, it was found that application of the blends of two incompatible polymers yields the transfer from the impermeable membrane structure to the membrane structure with reasonable flux (1 in Figure 8a).

It was found that addition of PEG-400 to the casting solution significantly increases membrane PWF (2 on Figure 8a,b) which is consistent with the data obtained in [26,27,28]. As was shown in the previous series of experiments, membranes formed from pristine 20 wt% PPSU and PSF solutions are not permeable for water at 0.1 MPa. At the same time, for PPSU:PSF ratios = (30–60):(70–40), PWF considerably increases up to 195 L∙m^−2^ h^−1^ when 15 wt% PEG-400 is added to the casting solution. When PEG-400 is introduced to casting solutions, the range of PPSU:PSF ratios suitable for preparation of high-flux membranes increases. Pure water fluxes of the membranes cast using PEG-400 are twice as high as those of the membranes cast from solutions not containing PEG-400 (Figure 8a).

It was found that the increase of PEG-400 concentration in the casting solution yields the dramatic increase of PWF of blend membranes (series C) (Figure 8b). It was shown that PWF reaches 270 L∙m^−2^ h^−1^, when PEG-400 concentration increases up to 20 wt% in the PPSU-PSF blend casting solution with the polymer ratio 50:50 (Figure 8b). Such an increase is reasonable, since PEG-400 reduces thermodynamic stability of the casting solution and prevents the formation of dense structure of the selective layer. It occurs due to the faster formation of polymer agglomerates which is attributed to the combination of the liquid-liquid and polymer-polymer phase separation upon membrane preparation via NIPS (Figure 7).

Membrane preparation from almost identical blend PPSU/PSF solutions in NMP was reported in [26]. The difference with the present study was that PEG with higher molecular weight (PEG-1000, M_n_ = 1000 g·mol^−1^) was used. This difference resulted in membranes with PWF not exceeding 20 L∙m^−2^ h^−1^ at 0.1 MPa. The PWF increase of PPSU membranes upon addition of PEG-1000 and a second polymer (PSF) was also observed [26]. However, maximum PWF was achieved at 70:30 PPSU:PSF ratio. Application of different molecular weights of PEG is likely to account for these differences due to the increase of casting solution viscosity when PEG-1000 is used instead of PEG-400.

**Figure 9:**
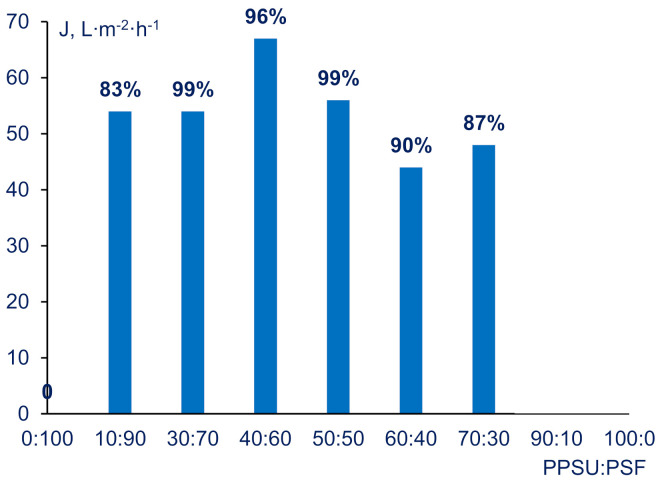
Human serum albumin (HSA) solution flux (J) and HSA rejection of membranes vs. PPSU:PSF blend ratio in casting solution (series A).

HSA solution flux and HSA rejection coefficient were determined for the membranes cast from 20 wt% PPSU/PSF solutions (series A). As shown on Figure 9, when PPSU:PSF ratios increase from 10:90 to 40:60, HSA solution flux increases. Further increase of PPSU content in the blend yields the gradual decrease in pure water flux. In the meantime, HSA rejection coefficients are within 83-99% range with the maximum at 30:70 and 50:50 PPSU:PSF ratios. A90, PSF100, and PPSU100 membranes were found to be impermeable for HSA solution. For C-50-15 and C-50-20 HSA solution fluxes were found to be 120 and 195 L∙m^−2^ h^−1^, respectively. The HSA rejections were determined to be 92% and 85%.

It is worth highlighting that achieved within this study pure water flux of 270 L∙m^−2^ h^−1^ and HSA rejection of 85% for C-50-20 PPSU/PSF blend membrane is a promising result that allows developing PPSU membranes for industrial applications.

## 4. Conclusions

This study is focused on the modification of PPSU membranes via introduction of the incompatible membrane forming polymer (PSF) to the casting solution to increase membrane performance in ultrafiltration. The objective of this work was to reveal the correlation between the properties (phase state, structure, and viscosity) of the blend PPSU and PSF solutions and properties (structure, performance in ultrafiltration) of porous anisotropic membranes.

PPSU and PSF were shown to be thermodynamically incompatible polymers. It was confirmed by the high degree of heterogeneity of their blend solutions in NMP. The highest degree of heterogeneity was observed when the blend contained commensurate amount of polymers, i.e., PPSU:PSF ratios = (30–60):(70–40). Higher degree of heterogeneity yields the higher precipitation rate of the polymer solution during the membrane formation via NIPS due to the combination of “liquid-liquid” phase separation upon the contact of polymer solution with the non-solvent and “polymer-polymer” phase separation due to the incompatibility of PPSU and PSF. It results in the formation of a more porous and less dense selective layer and an increase in membrane permeability. It was found that the usage of PPSU/PSF solutions with the highest degree of heterogeneity for membrane preparation yields the formation of membranes with the highest pure water flux. Application of PPSU/PSF blends as membrane forming polymers and PEG-400 as a pore-forming agent allowed reaching high pure water flux (270 L·m^−2^·h^−1^ at 0.1 MPa) maintaining human serum albumin rejection of 85%.

## Figures and Tables

**Table 1:** Compositions of the casting solution and PPSU/PSF blend membranes abbreviations.

Membrane Abbreviation	PPSU:PSF Weight Ratio	Casting Solution Compositions (wt.%)			
		PPSU	PSF	PEG-400	NMP
PSF100	0:100	0	20.0	0	80
A 10	10:90	2.0	18.0	0	80
A 20	20:80	4.0	16.0	0	80
A 30	30:70	6.6	13.3	0	80
A 40	40:60	8	12.0	0	80
A 50	50:50	10.0	10.0	0	80
A 60	60:40	12.0	8.0	0	80
A 70	70:30	13.3	6.6	0	80
A 80	80:20	16.0	4.0	0	80
A 90	90:10	18.0	2.0	0	80
PPSU100	100:0	20.0	0	0	80
PSF-PEG	0:100	0	20.0	15	65
B 10	10:90	2.0	18.0	15	65
B 20	20:80	4.0	16.0	15	65
B 30	30:70	6.6	13.3	15	65
B 40	40:60	8.0	12.0	15	65
B 50	50:50	10.0	10.0	15	65
B 60	60:40	12.0	8.0	15	65
B 70	70:30	13.3	6.6	15	65
B 80	80:20	16.0	4.0	15	65
B 90	90:10	18.0	2.0	15	65
PPSU-PEG	100:0	20.0	0	15	65
C50-5	50:50	10.0	10.0	5	75
C-50-10	50:50	10.0	10.0	10	70
C-50-13	50:50	10.0	10.0	13	67
C-50-15	50:50	10.0	10.0	15	65
C-50-18	50:50	10.0	10.0	18	62
C-50-20	50:50	10.0	10.0	20	60

**Table 2:** Coagulation values (CV) of polysulfone solutions in NMP.

Coagulant	CV of 1 wt% Solution of Polysulfones in NMP, g∙dL^−1^		
	PSF	PES	PPSU
Water	7.0	14.5	9.2
Glycerol	20.6	37.1	23.8
Ethylene glycol	26.7	39.5	30.9
Isopropanol	12.7	38.5	33.2
PEG-400	>250	>250	>330

## Data Availability

The data presented in this study are available on request from the corresponding author. At the time the project was carried out, there was no obligation to make the data publicly available.
